# Secretion of Galectin-9 as a DAMP during Dengue Virus Infection in THP-1 Cells

**DOI:** 10.3390/ijms18081644

**Published:** 2017-07-28

**Authors:** Isolde C. Dapat, Dyshelly Nurkartika Pascapurnama, Hiroko Iwasaki, Hannah Karen Labayo, Haorile Chagan-Yasutan, Shinichi Egawa, Toshio Hattori

**Affiliations:** 1 Division of International Cooperation for Disaster Medicine, International Research Institute of Disaster Science (IRIDeS), Tohoku Medical Megabank Organization, 2-1 Seiryo-machi, Aoba-ku, Sendai 980-8573, Japan; isoldedapat@med.tohoku.ac.jp (I.C.D.); dyshelly.nurkartika@gmail.com (D.N.P.); egawas@surg1.med.tohoku.ac.jp (S.E.); 2 Graduate School of Biomedical Engineering, Tohoku University, 6-6-05 Aramaki-aoba, Sendai 980-8579, Japan; hiwasaki@irides.tohoku.ac.jp; 3 Department of Virology, Graduate School of Medicine, Tohoku University, 2-1 Seiryo-machi, Aoba-ku, Sendai 980-8575, Japan; hkmlabayo@gmail.com; 4 Graduate School of Health Science Studies, Kibi International University, Iga-cho 8, Takahashi, Okayama 716-8508, Japan; haorile@foxmail.com

**Keywords:** galectin-9, *LGALS9*, DAMPs, dengue virus, THP-1

## Abstract

Damage-associated molecular patterns (DAMPs) are endogenous cellular molecules released to the extracellular environment in response to stress conditions such as virus infection. Galectins are β-galactoside-binding proteins that are widely expressed in cells and tissues of the immune system, are localized in the cell cytoplasm, and have roles in inflammatory responses and immune responses against infection. Elevated levels of galectin-9 (Gal-9) in natural human infections have been documented in numerous reports. To investigate the effect of dengue virus (DENV) infection on expression of endogenous Gal-9, monocytic THP-1 cells were infected with varying doses of DENV-3 (multiplicity of infection (MOI) 0.01, 0.03 and 0.1) and incubated at varying time points (Day 1, Day 2, Day 3). Results showed augmentation of Gal-9 levels in the supernatant, reduction of Gal-9 levels in the cells and decreased expression of *LGALS9* mRNA, while DENV-3 mRNA copies for all three doses remained stable through time. Dengue virus induced the secretion of Gal-9 as a danger response; in turn, Gal-9 and other inflammatory factors, and stimulated effector responses may have limited further viral replication. The results in this pilot experiment add to the evidence of Gal-9 as a potential DAMP.

## 1. Introduction

Dengue is a mosquito-borne viral disease of increasing incidence and expanding geographical range with an estimated 390 million infections every year [1] and 3.9 billion people in 128 countries at risk of infection [2]. It is caused by dengue virus, a member of the *Flaviviridae* family and is a small, enveloped virus that contains a single-stranded positive-sense RNA genome. It has four antigenically distinct, but closely related, serotypes (DENV-1 to DENV-4) that causes a range of diseases from a relatively benign, self-limiting dengue fever to severe, life-threatening dengue hemorrhagic fever and dengue shock syndrome [3].

Galectins are a family of sugar-binding proteins with one or two conserved carbohydrate recognition domains (CRDs) that have an affinity for β-galactosides. Galectin-9, one of 15 identified mammalian galectins, has a tandem-repeat type structure consisting of two distinct CRDs connected by a linker peptide of variable length. It has three isoforms based on the length of the linker peptide: short (14 amino acids), medium (26 amino acids) and long (58 amino acids) [4], generated from a single gene by alternative splicing [5]. Galectin-9 (gal-9) is encoded by the *LGALS9* gene. It was first reported as an eosinophil chemoattractant [6] then was found to have biological functions in cell aggregation, proliferation and survival, apoptosis, and immunomodulation of inflammation [7]. It is widely expressed in the liver, small intestine, thymus, kidney, spleen, lung, cardiac and skeletal muscle, [8] and in all cells of the immune system.

Galectins are synthesized and stored in the cytoplasm, but upon infection, cytosolic galectins are actively secreted by inflammatory activated cells. Galectins are considered as potential damage-associated molecular pattern (DAMP) or danger molecules which signal cell/tissue damage and elicit an effector response from immune cells [9]. Circulating Gal-9 levels were found to be elevated in the plasma or serum of patients with viral infections (reviewed in [10]) such as HIV [11], HCV [12], influenza A virus [13], and dengue virus [14,15], as well as in those with bacterial (tuberculosis) [16] and parasitic (malaria) [17] infections.

Monocytes, along with macrophages and dendritic cells, are the primary targets of dengue virus [18]. Here, we report the secretion of Gal-9 to the culture supernatant, and concomitant decrease in cell-associated Gal-9 and *LGALS9* expression in monocytic THP-1 cells in the presence of dengue virus serotype 3.

## 2. Results

### 2.1. Induction of Galectin-9 Secretion of THP-1 Cells by Dengue Virus Infection

**Figure 1:**
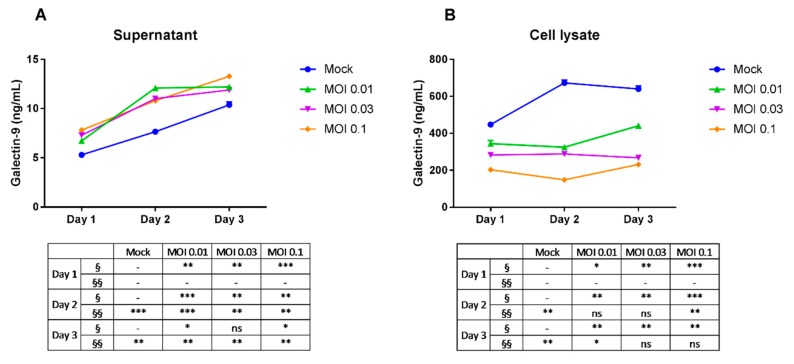
Galectin-9 levels in dengue virus-infected THP-1 cells. Cells were mock-infected or infected with dengue virus-3 (DENV-3) at varying doses. Cells were incubated and harvested after 1, 2 and 3 days post-infection. Extracellular (**A**) and intracellular (**B**) galectin-9 was assayed using ELISA. Unpaired *t*-test was used to calculate significance (ns = no significance, * *p* ≤ 0.05, ** *p* ≤ 0.01, *** *p* ≤ 0.001) vs. control. Data represent mean ± SEM (standard error of the mean) of representative data of three experiments with similar results. § and §§ represent dose- and time-dependent variables, respectively. MOI: multiplicity of infection.

To investigate the effect of dengue virus infection on endogenous Gal-9 secretion, we infected the THP-1 cell line with DENV-3. Cells were inoculated with varying doses of DENV-3 (multiplicity of infection (MOI) 0.01, MOI 0.03 and MOI 0.1), cultivated and harvested after 24, 48 and 72 h. Gal-9 levels in the supernatant and cell lysate of THP-1 cultures were measured by ELISA (enzyme-linked immunosorbent assay). Gal-9 was detected in THP-1 culture supernatants at higher levels in cultures infected with DENV-3 (from lowest dose of MOI 0.01 to highest of MOI 0.1) compared with mock-infected cells (Figure 1A). Mean Gal-9 levels for all three doses were highest on Day 3 post-infection and levels between doses were relatively similar. The significantly higher levels of Gal-9 present in the supernatant of DENV-3-infected cultures showed that dengue virus was able to induce Gal-9 secretion in THP-1 cells. The observed increase of the Gal-9 protein level in the mock group supernatant could be the result of cumulative amounts of basal levels of secreted Gal-9. Moreover, the THP-1 cell numbers in DENV-infected cultures were lower than in controls (data not shown) indicating that the differences in secreted Gal-9 protein were not due to an increased number of cells. The dose-relationship evaluation, which did not show significant changes of Gal-9 levels, signifies that the differences between the doses used may be too small to induce pronounced differences in Gal-9 secretion.

Gal-9 levels in the lysates of DENV-3 infected THP-1 cells were significantly lower than in the mock-infected cells for all three doses (Figure 1B) and the decrease is dose-dependent. The highest virus dose of MOI 0.1 showed the lowest Gal-9 levels for all incubation times. The significantly lower levels of Gal-9 present in the cell lysates of DENV-3 infected cultures indicated that dengue virus was able to suppress Gal-9 expression in THP-1 cells in a dose-dependent manner.

### 2.2. Reduction of Galectin-9 mRNA Expression in THP-1 Cells by Dengue Virus Infection

**Figure 2:**
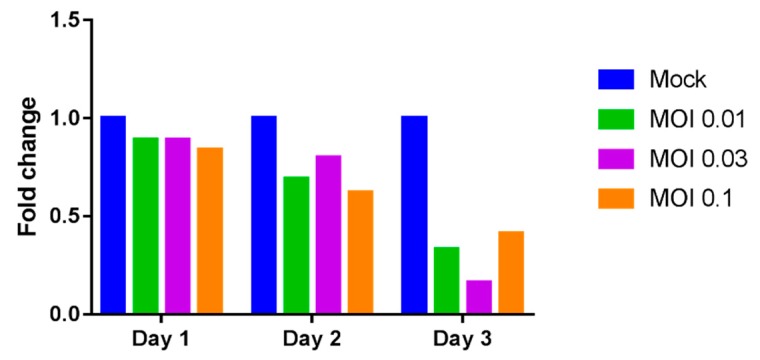
Relative expression of *LGALS9* mRNA. THP-1 cells were mock-infected or infected with DENV-3 at varying doses. Cells were incubated and harvested after 1, 2 and 3 days post-infection. Total RNA from cell lysates was extracted and reverse transcription quantitative RT-PCR was used to determine the expression of *LGALS9* mRNA. The mRNA levels of galectin-9 (Gal-9) were normalized to GAPDH mRNA.

A decreasing trend in the expression of *LGALS9* mRNA was observed in DENV-3 infected THP-1 cells as incubation time increased. Markedly lower levels of Gal-9 mRNA transcripts were detected on Day 3 in cells infected with DENV-3 at MOI 0.01 (3-fold decrease or 67% reduction), MOI 0.03 (6.25-fold decrease or 84% reduction) and MOI 0.1 (2.44-fold decrease or 59% reduction) compared to mock-infected controls (Figure 2). The reduced Gal-9 mRNA transcripts in infected THP-1 cells signified that the presence of dengue virus was able to suppress Gal-9 transcription in THP-1 cells in a time-dependent manner.

### 2.3. Replication of Dengue Virus

**Figure 3:**
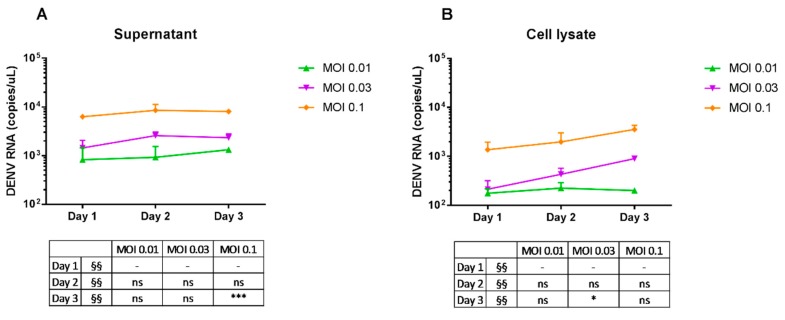
Dengue virus RNA replication in THP-1 cells. Cells were mock-infected or infected with DENV-3 at varying doses, incubated, and harvested after 1, 2 and 3 days post-infection. Total RNA from culture supernatant and cell lysates was extracted and reverse transcription quantitative real-time PCR was used to determine the expression of dengue virus mRNAs. Viral genome copy numbers in the supernatant (**A**) and cells (**B**) were estimated using dengue virus RNA as standards. Unpaired *t*-test was used to calculate significance (ns = no significance, * *p* ≤ 0.05 and *** *p* ≤ 0.001) vs. Day 1. §§ represents time-dependent variable.

Dengue virus mRNA in the supernatant and in the cells was measured by quantitative real-time PCR. Dengue virus particles in the supernatant (Figure 3A), as well as in the cells (Figure 3B), remained stable through time for the three doses, with mostly no significant differences in copy number with their respective MOIs at Day 1. The stable levels of DENV-3 mRNA transcripts showed that, in general, there was no increased viral replication in infected THP-1 cultures.

## 3. Discussion

Damage-associated molecular patterns (DAMPs) are cellular molecules secreted into the extracellular environment under stress conditions such as viral infection. Upon infection, cells release these molecules into the extracellular milieu to facilitate a variety of cellular responses including recruitment of innate immune cells, activation of cytokines and chemokines, and binding to the infecting pathogen to serve as receptors for phagocytosis and apoptosis [19].

The function of Gal-9 as DAMPs in human dengue virus infection possibly includes modulation of immune cells. Our results showed higher Gal-9 levels with lower cell numbers in DENV-infected THP-1 cells compared with controls, which is similar to our previous results that showed a negative correlation between Gal-9 plasma levels and monocyte percentages [14]. This negative correlation suggests a role for Gal-9 in the migration of monocytes and adherence to the inflamed endothelial cells during human dengue virus infections. The released Gal-9 may further activate monocytes in an autocrine manner. In addition, extracellular Gal-9 is a potent chemoattractant for eosinophils and possibly promotes eosinophil migration during dengue virus infection. Moreover, Gal-9 has been reported to be an epithelial polarity regulator and induce fatal autophagy in cancer cells [20], and was proposed to participate in immunopathology of pleural effusion in pulmonary tuberculosis [21]. These findings further support the involvement of Gal-9 in plasma leakage in DENV infection [14] and in host response dynamics, in addition to its role as an immunomodulator.

Similar to our findings in THP-1 cells, Gal-9 secretion has been demonstrated in several cell lines [4,22], and Gal-9 induction and release has been observed in natural human infection by dengue virus [14]. Galectins are localized in the cell cytoplasm and in the nucleus, or attached to the extracellular matrix [23,24]. Secretion of galectins occur via a non-classical pathway, as galectins lack a signal sequence for conventional extracellular transport via the endoplasmic reticulum (ER)-Golgi pathway [25].

Gal-9 is highly expressed in cells of the immune system, and THP-1—a monocyte line—expresses Gal-9 constitutively [4]. We found that infection of THP-1 cells with DENV-3 resulted in the augmentation of secreted levels of the protein in the supernatant while intracellular Gal-9 levels decreased. The infected THP-1 cells released more Gal-9 to the culture supernatant, which could partly explain the reduction of cell-associated Gal-9 protein after infection. The reduction of Gal-9 in cells could also be attributed to the suppressed expression of Gal-9 mRNA. THP-1 cells infected with DENV-3 have lower *LGALS9* expression relative to that of cells grown in medium only. 

Previous reports have shown that dengue virus infection induced increased expression of galectin-9 at the cell-based protein or mRNA level [26,27]. The increased levels of Gal-9 we observed in the supernatant may be more of a change in Gal-9 localization, from inside the cell to the extracellular milieu, rather than an increase in protein translation as a response to dengue virus infection. This is supported by the observation of the decrease in intracellular Gal-9, as well as a decrease in *LGALS9* expression, after infection. The possibility of the involvement of the factors affecting Gal-9 transcription that led to the time-dependent decrease in *LGALS9* expression cannot be completely excluded.

Dengue virus infection triggers the release of inflammatory cytokines, chemokines, immune complexes and other inflammatory mediators. We previously reported the increase in 16 cytokines and chemokines in dengue patients during the critical phase of infection [14], as well as the induction of osteopontin—a multifunctional extracellular matrix protein implicated in the pathogenesis of various inflammatory disorders—in DENV-3 infected THP-1 cells [28]. Gal-9 itself activates inflammatory cytokines in monocytes [29] and enhances cytokine secretion in the human mast cell line [30]. Kurane and Ennis [31] found that interferon (IFN)-α in the culture fluids of dengue virus-infected human monocytes inhibited infection of human monocytes by the virus, thereby limiting viral replication. We found stable levels of DENV-3 in the supernatant of THP-1 cultures for up to 72 h for all three infection doses. Cytokines and other factors secreted into the supernatant may have inhibited dengue virus infection of THP-1 cells, and production and/or release of new virus progeny. 

Dengue virus is an enveloped virus with a lipid membrane. There are 180 identical copies of the major envelope glycoprotein (E) protein attached to the surface of the viral membrane. Glycans, through binding with the two *N*-glycosylation sites on E protein, have been implicated in cellular attachment and viral entry [32,33]. Studies on other galectins found that exogenous recombinant Gal-1 in dengue virus-infected ECV-304 cells inhibited virus production by directly binding to the dengue virus and inhibiting viral adsorption and internalization to the cells [34]. Recombinant Gal-1 also inhibited influenza A virus infection by direct binding to the envelope glycoproteins and inhibiting hemagglutination activity and infectivity [35]. Gal-1 is expressed ubiquitously in human tissues and cells, including immune cells, and it may have contributed to the limitation of dengue virus replication in monocytic THP-1 cells. Similarly, Gal-9 may bind to dengue virus via the interaction of its CRDs and viral glycans, and limit dengue virus attachment to THP-1 cells.

Previous reports have demonstrated the induction of inflammatory factors and stimulation of effector responses that dengue virus elicited in THP-1 cells, and their possible roles in cell or immune activation and cell migration. THP-1 cells infected with dengue virus induced elevated levels of dengue hemorrhagic fever (DHF)-associated immunomediators such as interleukin (IL)-6, IL-8, interferon γ-induced protein-10 (IP-10), tumor necrosis factor alpha (TNFα) or IFNγ [36]. These immunomediators secreted in the supernatants of DENV-infected monocytes increased human microvascular endothelial cell (HMVEC) permeability and expression of adhesion molecules such as intercellular adhesion molecule 1 (ICAM-1), vascular cell adhesion molecule 1 (VCAM-1) and E-selectin [37]. In humans, dengue virus infection activates monocytes and the release of immunomodulatory cytokines, such as TNFα, from activated monocytes then contributes to increased permeability across endothelial cells and the hallmark vascular leak syndrome in more severe dengue cases [38].

The downregulation of *LGALS9* mRNA during DENV-3 infection is a novel finding and warrants additional investigation. This study is a pilot examination of the effect of dengue virus infection on galectin-9 expression in monocytic THP-1 cells. The results in this study add to the evidence of the role of galectins as potential damage-associated molecular patterns during acute viral infections such as dengue. The role of Gal-9 in the stimulation of effector responses and the subsequent inhibition of dengue viral replication needs to be elucidated.

## 4. Materials and Methods 

### 4.1. Cell Line and Virus

Human monocytic THP-1 cell line was obtained from the American Type Culture Collection (Manassas, VA, USA) and maintained in Roswell Park Memorial Institute (RPMI) 1640 medium (Wako Pure Chemical Industries, Osaka, Japan) supplemented with 10% heat-inactivated fetal bovine serum (FBS) (Thermo Fisher Scientific, Waltham, MA, USA). Cells were cultured in a humidified incubator under 5% CO_2_ at 37 °C.

Infected culture fluid virus stocks of dengue virus serotype 3 (DENV-3), isolated from a patient with dengue fever at San Lazaro Hospital in Manila, Philippines [14], were used to infect THP-1 cultures. Viral titer was determined previously by plaque assay using Vero cells, expressed as plaque-forming units per milliliter (pfu/mL), and was used to calculate the multiplicity of infection (MOI).

### 4.2. Infection with Dengue Virus (DENV)

THP-1 cells were seeded in a Nunc cell culture tube (Thermo Fisher Scientific, Waltham, MA, USA) at 2 × 10^5^ cells/tube in 1 mL of growth medium supplemented with FBS and incubated overnight. Tubes were centrifuged at 1200 rpm for 5 min and the spent medium was decanted; 100 µL of DENV-3 diluted in culture medium was added to each tube at final MOIs of 0.01, 0.03, and 0.1. The tubes were incubated for 1.5 h with rocking every 30 min to ensure virus adsorption. Cells were washed twice with medium and cultured in 1 mL of fresh medium supplemented with 2% FBS before they were harvested on days 1, 2, and 3 postinfection.

### 4.3. Collection of DENV-Infected Cells and RNA Extraction

THP-1 cells were collected by centrifugation at 1200 rpm for 5 min and the supernatant was transferred to 1.5 mL tubes (Eppendorf, Hamburg, Germany). Cells were lysed with lysis buffer (50 mL Pierce M-PER Mammalian Protein Extraction Reagent, 1 tablet of Roche Protease Inhibitor Cocktail, 10 mM Lactose Monohydrate) or homogenized with 200 µL of homogenization solution from the Maxwell^®^ 16 LEV simplyRNA Cells kit (Promega, Madison, WI, USA). Total RNA was extracted using Maxwell^®^ 16 LEV simplyRNA Cells kit in a Promega Maxwell^®^ 16 Instrument according to the manufacturer’s instructions. RNA samples were stored at −80 °C.

### 4.4. Determination of Gal-9 Concentration by Enzyme-Linked Immunosorbent Assay (ELISA)

Galectin-9 levels in the cell culture supernatant and cell lysate were quantified using the Human Galectin-9 DuoSet^®^ ELISA Development System (R&D Systems, Minneapolis, MN, USA) according to the manufacturer’s instructions and as described previously [39]. The supernatant was diluted 2-fold, THP-1 cell lysates 50-fold. 

### 4.5. Determination of Relative Galectin-9 mRNA Expression by RT-PCR

A forward and reverse primer set (LGALS9-F, 5′-GAAATGACATTGCCTTCCACTTCA-3′ and LGALS9-R, 5′-GAAGAGGATCCCGTTCACCA-3′) and a Taqman probe, labeled at the 5′ end with 6-carboxyfluorescein (FAM) reporter and at the 3′ end with Black Hole quencher (BHQ)-1, LGALS9-P, 5′-[FAM]-CAGCTTCCGTTCTGCCTCGTGTTGC-[BHQ1]3′ (Takara Bio, Kusatsu, Japan) were used to quantify *LGALS9* mRNA (accession number NM_002308). Glyceraldehyde 3-phosphate dehydrogenase (GAPDH) was used as a reference gene (accession number NM_002046) for normalization of expression levels and was amplified with the following forward primer GAPDH-F, 5′-GCACCGTCAAGGCTGAGAAC-3′, reverse primer GAPDH-R, 5′-TGGTGAAGACGCCAGTGGA-3′ and Taqman probe, 5′[FAM]-TCCACGACGTACTCAGCGCCAGCAT-[BHQ1]3′. The mean fold change in *LGALS9* mRNA level was calculated as fold change = 2^−∆∆*C*t^ [40].

### 4.6. Determination of DENV Copy Number by Reverse Transcription Quantitative Real-Time PCR

Viral copy numbers in culture supernatants and cell lysates were measured by qRT-PCR using the RNA UltraSense One-Step Quantitative RT-PCR System (Invitrogen, Carlsbad, CA, USA) and Thermal Cycler Dice Real Time System (Takara Bio, Otsu, Japan) according to the manufacturers’ protocols and as described previously [14]. Primers and fluorescent probes specific to the 3′ untranslated region of each of the four DENV genotypes have been published previously [14]. The forward and reverse primers were as follows: 5′-AAGGACTAGAGGTTAGAGGAGACCC-3′ and 5′-CGTTCTGTGCCTGGAATGATG-3′, and the TaqMan probe, 5′[FAM]-TGGGARAGACCAGAGATCCTGCTGTCT-[BHQ1]3′ [41]. 

### 4.7. Statistical Analysis

The comparisons between data groups were performed with the two-tailed *t*-test in GraphPad Prism version 7.00 for Windows (GraphPad Software, La Jolla, San Diego, CA, USA, Available online: www.graphpad.com). A *p*-value of less than 0.05 was considered significant.

## References

[1] Bhatt S., Gething P.W., Brady O.J., Messina J.P., Farlow A.W., Moyes C.L., Drake J.M., Brownstein J.S., Hoen A.G., Sankoh O. (2013). The global distribution and burden of dengue. Nature.

[2] Brady O.J., Gething P.W., Bhatt S., Messina J.P., Brownstein J.S., Hoen A.G., Moyes C.L., Farlow A.W., Scott T.W., Hay S.I. (2012). Refining the global spatial limits of dengue virus transmission by evidence-based consensus. PLoS Negl. Trop. Dis..

[3] World Health Organization. Dengue and Severe Dengue.

[4] Chabot S., Kashio Y., Seki M., Shirato Y., Nakamura K., Nishi N., Nakamura T., Matsumoto R., Hirashima M. (2002). Regulation of galectin-9 expression and release in Jurkat T cell line cells. Glycobiology.

[5] Lipkowitz M.S., Leal-Pinto E., Rappoport J.Z., Najfeld V., Abramson R.G. (2001). Functional reconstitution, membrane targeting, genomic structure, and chromosomal localization of a human urate transporter. J. Clin. Investig..

[6] Hirashima M. (2000). Ecalectin/galectin-9, a novel eosinophil chemoattractant: Its function and production. Int. Arch. Allergy Immunol..

[7] Matsumoto R., Hirashima M., Kita H., Gleich G.J. (2002). Biological activities of ecalectin: A novel eosinophil-activating factor. J. Immunol..

[8] Wada J., Ota K., Kumar A., Wallner E.I., Kanwar Y.S. (1997). Developmental regulation, expression, and apoptotic potential of galectin-9, a β-galactoside binding lectin. J. Clin. Investig..

[9] Sato S., St-Pierre C., Bhaumik P., Nieminen J. (2009). Galectins in innate immunity: Dual functions of host soluble β-galactoside-binding lectins as damage-associated molecular patterns (DAMPs) and as receptors for pathogen-associated molecular patterns (PAMPs). Immunol. Rev..

[10] Merani S., Chen W., Elahi S. (2015). The bitter side of sweet: The role of galectin-9 in immunopathogenesis of viral infections. Rev. Med. Virol..

[11] Chagan-Yasutan H., Saitoh H., Ashino Y., Arikawa T., Hirashima M., Li S., Usuzawa M., Oguma S., EF O.T., Obi C.L. (2009). Persistent elevation of plasma osteopontin levels in hiv patients despite highly active antiretroviral therapy. Tohoku J. Exp. Med..

[12] Harwood N.M., Golden-Mason L., Cheng L., Rosen H.R., Mengshol J.A. (2016). HCV-infected cells and differentiation increase monocyte immunoregulatory galectin-9 production. J. Leukoc. Biol..

[13] Katoh S., Ikeda M., Shimizu H., Mouri K., Obase Y., Kobashi Y., Fukushima K., Hirashima M., Oka M. (2014). Increased levels of plasma galectin-9 in patients with influenza virus infection. Tohoku J. Exp. Med..

[14] Chagan-Yasutan H., Ndhlovu L.C., Lacuesta T.L., Kubo T., Leano P.S., Niki T., Oguma S., Morita K., Chew G.M., Barbour J.D. (2013). Galectin-9 plasma levels reflect adverse hematological and immunological features in acute dengue virus infection. J. Clin. Virol..

[15] Liu K.T., Liu Y.H., Chen Y.H., Lin C.Y., Huang C.H., Yen M.C., Kuo P.L. (2016). Serum galectin-9 and galectin-3-binding protein in acute dengue virus infection. Int. J. Mol. Sci..

[16] Shiratori B., Zhao J., Okumura M., Chagan-Yasutan H., Yanai H., Mizuno K., Yoshiyama T., Idei T., Ashino Y., Nakajima C. (2016). Immunological roles of elevated plasma levels of matricellular proteins in japanese patients with pulmonary tuberculosis. Int. J. Mol. Sci..

[17] Dembele B.P., Chagan-Yasutan H., Niki T., Ashino Y., Tangpukdee N., Shinichi E., Krudsood S., Kano S., Hattori T. (2016). Plasma levels of galectin-9 reflect disease severity in malaria infection. Malar. J..

[18] Kou Z., Quinn M., Chen H., Rodrigo W.W., Rose R.C., Schlesinger J.J., Jin X. (2008). Monocytes, but not T or B cells, are the principal target cells for dengue virus (DV) infection among human peripheral blood mononuclear cells. J. Med. Virol..

[19] Schaefer L. (2014). Complexity of danger: The diverse nature of damage-associated molecular patterns. J. Biol. Chem..

[20] Wiersma V.R., de Bruyn M., Wei Y., van Ginkel R.J., Hirashima M., Niki T., Nishi N., Zhou J., Pouwels S.D., Samplonius D.F. (2015). The epithelial polarity regulator LGALS9/galectin-9 induces fatal frustrated autophagy in KRAS mutant colon carcinoma that depends on elevated basal autophagic flux. Autophagy.

[21] Zhao J., Shiratori B., Chagan-Yasutan H., Matsumoto M., Niki T., Tanaka M., Takahashi Y., Usami O., Ashino Y., Hattori T. (2017). Secretion of IFN-γ associated with galectin-9 production by pleural fluid cells from a patient with extrapulmonary tuberculosis. Int. J. Mol. Sci..

[22] Niki T., Tsutsui S., Hirose S., Aradono S., Sugimoto Y., Takeshita K., Nishi N., Hirashima M. (2009). Galectin-9 is a high affinity IgE-binding lectin with anti-allergic effect by blocking IgE-antigen complex formation. J. Biol. Chem..

[23] Moutsatsos I.K., Wade M., Schindler M., Wang J.L. (1987). Endogenous lectins from cultured cells: Nuclear localization of carbohydrate-binding protein 35 in proliferating 3T3 fibroblasts. Proc. Natl. Acad. Sci. USA.

[24] Sato S., Burdett I., Hughes R.C. (1993). Secretion of the baby hamster kidney 30-kDa galactose-binding lectin from polarized and nonpolarized cells: A pathway independent of the endoplasmic reticulum-golgi complex. Exp. Cell Res..

[25] Delacour D., Koch A., Jacob R. (2009). The role of galectins in protein trafficking. Traffic.

[26] Warke R.V., Xhaja K., Martin K.J., Fournier M.F., Shaw S.K., Brizuela N., de Bosch N., Lapointe D., Ennis F.A., Rothman A.L. (2003). Dengue virus induces novel changes in gene expression of human umbilical vein endothelial cells. J. Virol..

[27] Hsu Y.L., Wang M.Y., Ho L.J., Huang C.Y., Lai J.H. (2015). Up-regulation of galectin-9 induces cell migration in human dendritic cells infected with dengue virus. J. Cell. Mol. Med..

[28] Pascapurnama D.N., Labayo H.K., Dapat I., Nagarajegowda D.D., Zhao J., Zhang J., Yamada O., Kikuchi H., Egawa S., Oshima Y. (2017). Induction of osteopontin by dengue virus-3 infection in THP-1 cells: Inhibition of the synthesis by brefelamide and its derivative. Front. Microbiol..

[29] Matsuura A., Tsukada J., Mizobe T., Higashi T., Mouri F., Tanikawa R., Yamauchi A., Hirashima M., Tanaka Y. (2009). Intracellular galectin-9 activates inflammatory cytokines in monocytes. Genes Cells.

[30] Kojima R., Ohno T., Iikura M., Niki T., Hirashima M., Iwaya K., Tsuda H., Nonoyama S., Matsuda A., Saito H. (2014). Galectin-9 enhances cytokine secretion, but suppresses survival and degranulation, in human mast cell line. PLoS ONE.

[31] Kurane I., Ennis F.A. (1988). Production of interferon α by dengue virus-infected human monocytes. J. Gen. Virol..

[32] Hung S.L., Lee P.L., Chen H.W., Chen L.K., Kao C.L., King C.C. (1999). Analysis of the steps involved in dengue virus entry into host cells. Virology.

[33] Modis Y., Ogata S., Clements D., Harrison S.C. (2005). Variable surface epitopes in the crystal structure of dengue virus type 3 envelope glycoprotein. J. Virol..

[34] Toledo K.A., Fermino M.L., Andrade Cdel C., Riul T.B., Alves R.T., Muller V.D., Russo R.R., Stowell S.R., Cummings R.D., Aquino V.H. (2014). Galectin-1 exerts inhibitory effects during DENV-1 infection. PLoS ONE.

[35] Yang M.L., Chen Y.H., Wang S.W., Huang Y.J., Leu C.H., Yeh N.C., Chu C.Y., Lin C.C., Shieh G.S., Chen Y.L. (2011). Galectin-1 binds to influenza virus and ameliorates influenza virus pathogenesis. J. Virol..

[36] Kelley J.F., Kaufusi P.H., Volper E.M., Nerurkar V.R. (2011). Maturation of dengue virus nonstructural protein 4B in monocytes enhances production of dengue hemorrhagic fever-associated chemokines and cytokines. Virology.

[37] Kelley J.F., Kaufusi P.H., Nerurkar V.R. (2012). Dengue hemorrhagic fever-associated immunomediators induced via maturation of dengue virus nonstructural 4B protein in monocytes modulate endothelial cell adhesion molecules and human microvascular endothelial cells permeability. Virology.

[38] Durbin A.P., Vargas M.J., Wanionek K., Hammond S.N., Gordon A., Rocha C., Balmaseda A., Harris E. (2008). Phenotyping of peripheral blood mononuclear cells during acute dengue illness demonstrates infection and increased activation of monocytes in severe cases compared to classic dengue fever. Virology.

[39] Chagan-Yasutan H., Lacuesta T.L., Ndhlovu L.C., Oguma S., Leano P.S., Telan E.F., Kubo T., Morita K., Uede T., Dimaano E.M. (2014). Elevated levels of full-length and thrombin-cleaved osteopontin during acute dengue virus infection are associated with coagulation abnormalities. Thromb. Res..

[40] Livak K.J., Schmittgen T.D. (2001). Analysis of relative gene expression data using real-time quantitative pcr and the 2^−ΔΔ*C*t^ method. Methods.

[41] Warrilow D., Northill J.A., Pyke A., Smith G.A. (2002). Single rapid TaqMan fluorogenic probe based PCR assay that detects all four dengue serotypes. J. Med. Virol..

